# Paravalvular Leakages after Surgical Aortic-Valve Replacement and after Transcatheter Aortic-Valve Implantation: Strategies to Increase the Success Rate of Percutaneous Closure

**DOI:** 10.3390/jcm11112989

**Published:** 2022-05-25

**Authors:** Xavier Freixa, Rami Gabani, Pedro Cepas-Guillén, Eduardo Flores-Umanzor, Rodrigo Estévez-Loureiro, Eustaquio Maria Onorato

**Affiliations:** 1 Interventional Cardiology Department, Hospital Clinic of Barcelona, University of Barcelona, 08036 Barcelona, Spain; xavierfreixa@hotmail.com (X.F.); gabani@clinic.cat (R.G.); cepas@clinic.cat (P.C.-G.); eduardo.floresumanzor@uhn.ca (E.F.-U.); 2 Cardiology Department, Alvaro Cunqueiro University Hospital, 36213 Vigo, Spain; roiestevez@hotmail.com; 3 Interventional Cardiology Department, Centro Cardiologico Monzino, Istituto di Ricovero e Cura a Carattere Scientifico (IRCCS), 20138 Milan, Italy

**Keywords:** paravalvular leak, transcatheter closure, surgical aortic-valve replacement, transcatheter, aortic-valve replacement, paravalvular-leak regurgitation

## Abstract

Moderate to severe paravalvular-leak (PVL) regurgitation after surgical aortic-valve replacement or after transcatheter valve implantation represents a well-known complication associated with symptoms related to heart failure, hemolysis, or both in patients with multiple comorbidities and with poor prognostic outcomes. The transcatheter closure of aortic paravalvular leaks (APVLs) is currently considered a valid alternative to cardiac surgery. Nevertheless, careful patient selection, optimal cardiac imaging for intraprocedural guidance, and expert operators are key for success. Although technically demanding, particularly in APVLs after transcatheter valve implantation, catheter-based closure is an effective, less invasive, and often the only option for high-risk patients with symptomatic PVL regurgitation.

## 1. Introduction

The percutaneous treatment of APVLs after conventional surgical aortic-valve replacement (AVR) or after transcatheter valve implantation (TAVI) is currently considered a valid alternative to cardiac surgery [1]. The clinical importance of APVLs arises from the evidence that they have a deleterious effect on ventricular function and lead to increased mortality [2]. Therefore, there is a need for the timely treatment of PVLs.

## 2. Materials and Methods

This viewpoint is based on the author’s personal experiences in the field of structural and adult congenital interventional cardiology, particularly in transcatheter PVL closure over 10 years, X.F.R. as a co-author of Spanish Real World PVLs Closure Registry [3], and E.M.O. as the principal investigator and coordinator of the International Multicenter PLD Registries [4,5] with a cohort of more than 200 patients, and from their knowledge of the literature and other experts’ experience through regular case discussions.

## 3. Imaging Approach

Cardiac imaging, especially two- and three-dimensional transthoracic/transesophageal echocardiography (2D/3D TTE/TEE) and cardiac angiography play an essential role in the diagnosis, guidance of intervention and subsequently in the evaluation of the outcomes of the procedure.

PVLs are studied in multiple angles to document their geometry. For instance, to visualize APVLs located in the right sinus of Valsalva, fluoroscopy will be oriented in the left anterior oblique view, those located in the left coronary sinus will be in the right anterior oblique view, and non-coronary PVLs will be in the lateral view. Furthermore, fluoroscopy helps to differentiate intraprosthetics from PVL regurgitation by looking at the closing and opening prosthetic angles [6].

TTE is very useful in identifying the APVL, particularly when the leakage is in the anterior position, as the posterior region is hidden by reverberation produced by the prosthetic annulus. The optimal views for the detection of regurgitant jets include the parasternal long axis, parasternal short axis, apical long axis, and five-chamber views. In the parasternal-short-axis view, the color flow Doppler interrogation of the sewing ring may be able to localize and define the extent of the leakage. TTE is also used to represent APVLs in a clockwise fashion [7]. This is a very useful system to follow up the PVL regurgitation. Nevertheless, it is noteworthy to mention that the TTE o’clock representation of the Valsalva aortic sinus does not correspond to TEE or to the surgical-view representation, and this must be taken into account in order to avoid misinterpretations.

2D/3D intracardiac echocardiography (ICE) represents an alternative or a complementary technique to TEE; it is exclusively indicated for procedural guidance, providing a high image resolution that is useful for very sick patients in whom local anesthesia may be more desirable.

Additional baseline image techniques include multidetector computed tomography angiography (MDCTA) with 3D or four-dimensional (4D) reconstruction and cardiac magnetic resonance (CMR), which are able to visualize the PVL anatomy more accurately, examining the entire circumference of the prosthetic ring on the axial oblique image plane performing precise flow-imaging and volume-based measurements. Furthermore, MDCT can provide extracardiac and coronary assessment that represents valuable help in determining the procedural risk to the patients.

The integration of computed tomography with fluoroscopy (CT–fluoroscopy fusion) or echocardiography with fluoroscopy (echo–fluoroscopy fusion) in the catheterization laboratory allows operators to overcome many of the challenges posed when these imaging modalities are used individually. In particular, the novel software technology, the EchoNavigator^®^system (Philips Healthcare, Best, The Netherlands) allows unique real-time fusion of live X-ray and live echo images for intuitive guidance during PVL-closure procedures, providing live dynamic imaging and allowing the interventionalist to view catheters and devices (by fluoroscopy) and soft-tissue anatomy (by echocardiography) in the same image and orientation. The incremental value of fusion modalities is key in cases of fluoroscopically invisible bioprosthesis or anatomically hard-to-approach calcified serpiginous tracts.

Nowdays, there is also the potential of 3D cardiac modeling for a thorough evaluation of the anatomical structure of these defects and their relationship to adjacent structures. Furthermore, 3D-printed modeling allows for testing the device before the interventional procedure, thereby limiting residual leaks and preventing serious complications.

Actually, APVLs are significantly different compared to mitral PVLs in terms of both assessment and treatment strategies. Firstly, the location of APVLs is determined by the aortic sinus position (left coronary, right coronary and non-coronary sinus) more than the mitral-valve clockface used in mitral PVLs. Secondly, angiographic contrast injection within the affected aortic sinus might provide more anatomic information in terms of PVL location, trajectory and size than both TTE/TEE. Thirdly, since additional PVL information provided by TEE as compared to TTE or angiography is limited, less invasive approaches without general anesthesia might be considered.

## 4. PVL Regurgitation after AVR: Procedural Aspects

In contrast to mitral leakages, post-AVR APVLs require arterial access and most cases do not need an AV loop. General anesthesia can be used, but moderate procedural sedation without intubation is also acceptable. APVLs are usually crossed in a retrograde way from the aorta to the left ventricle (LV), requiring an arterial access large enough to accommodate the delivery sheath. For this reason, APVLs have been classically approached through the femoral artery with 6- to 8-Fr introducers. However, the evolution of delivery catheters and closure devices currently allow the implantation of small, medium and large devices through 4-Fr, 5-Fr and 6-Fr, respectively, which opens the possibility to treat APVLs through the radial artery. In fact, they can be effectively and safely treated in a minimally invasive approach, consisting of mild sedation instead of general anesthesia, angiographic and TTE instead of TEE guidance, and a radial instead of a femoral approach [8]. The benefit of the radial over the femoral access has been demonstrated in PCI [9]. In fact, the need for early restoration of anticoagulation after PVL treatment in patients with mechanical valves can increase this benefit as these patients present a higher risk of vascular complications [10]. Other relevant technical considerations are the need for at least 90 cm, but ideally 110 cm, delivery catheters as the standard 60 to 80 cm catheters are generally not long enough to cross the PVL through the radial access. Alternatively, a left subclavian-artery approach (hybrid technique) could also be considered in patients with severe tortuosity of the aorto-iliac segment and the common iliac arteries or with a very horizontal ascending aorta. The shorter distance between the access site and the aortic valve is even seen as a potential technical advantage because of the short distance between the delivery catheter and the annulus.

Comprehensive knowledge of the inner diameters of the delivery catheters is required in order to identify the largest device that can progress through every catheter.

Transcatheter PVL closure was initially performed as an “off-label” indication with different occluders that have been created for other applications such as atrial septal, ventricular septal or patent ductus arteriosus closure. Those devices are not typically recommended for PVL cases due to several technical drawbacks and shortcomings (larger transcatheter delivery profile, bulkier design, lack of correct sizes, appropriate shapes, or proper waist configurations).

The AMPLATZER™ Vascular Plug (AVP, Abbott) family is the most frequently used technology in the United States for PVL closure as plugs can progress through smaller catheters compared to other devices with the same size. In general, the AVP 3 is the preferred closure device for most operators [3]. Other devices such as the AVP 4 are also used when conventional delivery catheters cannot progress through the leak. In summary, any AVP 3 device including the largest size (14 × 5 mm) can be advanced through delivery catheters with a minimum inner diameter >2 mm, such as a 6-Fr Destination (Terumo), 6-Fr Flexor (Cook) or any 7-Fr Guiding catheter. In the absence of progression of the previous catheters, catheters with a minimum inner diameter >1.8 mm such as a 5-Fr Destination (Terumo), 5-Fr Flexor (Cook) or any 6-Fr Guiding catheter would allow the implantation of AVP 3-10 × 5 mm or smaller. Finally, for very challenging paths or small leaks in which only 5-Fr or 4-Fr diagnostic catheters can be crossed (minimum inner diameter >1.3 mm), there is always the possibility to advance an AVP 4–8 mm.

**Figure 1:**
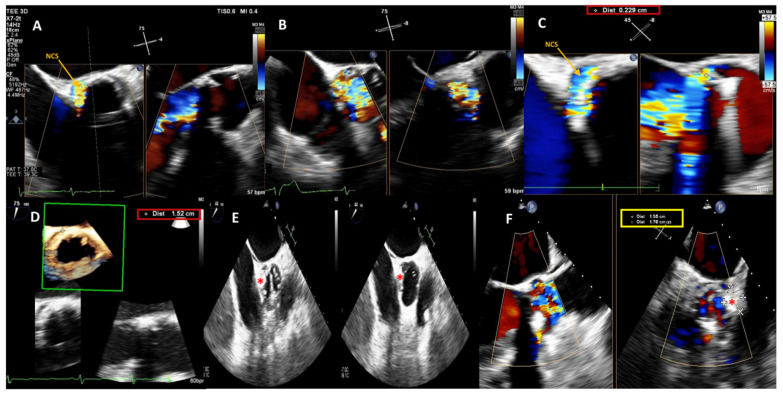
Baseline 2D/3D Transesophageal Echocardiogram (TEE) color Doppler showing a 15 × 2.3 mm large crescent-shaped NCS paravalvular leak with severe regurgitant jet (**A**–**C**) and 3D TEE color Doppler showing the PVL dimensions and location (**D**). Post-procedure 2D TEE color Doppler showing the correct position of the device (red star) during the cardiac cycle (diastolic and systolic frames, respectively) (**E**) and confirming stability of the 14 × 6 mm rectangular waist PLD (red star) without impingement on the mechanical prosthetic aortic valve (**F**). NCS, non-coronary sinus; PLD, Occlutech Paravalvular Leak device.

**Figure 2:**
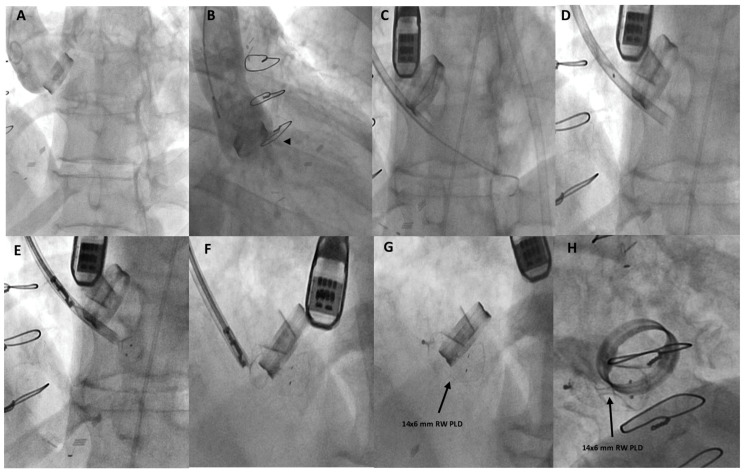
Fluoro-angiographic procedural steps. Ascending-aorta angiography by a 6-Fr pigtail catheter from the right femoral artery showing paravalvular-leak regurgitation (black arrowhead) (**A**,**B**); from left subclavian-artery approach (surgically exposed) a 9-Fr delivery sheath was advanced over the 0.035–260 cm stiff guidewire placed in the left ventricle (**C**); the distal and proximal discs of the 14 × 6 mm rectangular waist (RW) PLD were then step-by-step advanced (**D**–**F**) while still anchored to the delivery system; correct and stable position of the deployed PLD (black arrows) (**G**,**H**).

Alternatively, the Occlutech Paravalvular Leak Device (PLD) (Occlutech, Helsingborg, Sweden), a self-expanding, flexible, double-disc device made from nitinol-braided wires, is a specifically designed device, certified in Europe for the treatment of mitral and aortic leaks. Two different disc geometries are available, square and rectangular, connected by a waist of 19 different sizes and 4 different shapes to improve stability and sealing. The PLD has a special braiding that avoids a distal hub, giving flexibility and adaptability with a high success rate in achieving complete closure. In cases with large crescent-shaped defects requiring multiple vascular plugs, the implantation of a single large rectangular device to the edges increases the success rate and lowers residual regurgitation without interfering with the valve (Figure 1 and Figure 2, Supplementary Figures S1 and S2) [4,5,11,12,13].

Due to the morphological heterogeneity of PVL defects, operators must decide on the most compatible device. With a comprehensive preprocedural diagnostic assessment (2D/3D TEE and MDCT with its superior anatomical characterization and multiplanar formatting), operators can determine the location, size, extent, defect course, and ideal closure device to optimize outcomes and minimize complications such as device embolization. Regarding APVLs both post-cAVR or after TAVI, in our practice, the device choice may vary depending on each individual case anatomy. For small and very tortuous and elongated tracks, the AVP has some advantages over the PLD due to the fact that smaller delivery catheters and long sheaths are required. The PLD should be taken into consideration when the leak/leaks are crescentic and larger. In that case, one single PLD would probably be a better option.

The treatment strategy for APVLs is frequently the same. After obtaining radial or femoral access, a 4-Fr or 5-Fr diagnostic catheter is advanced. Some operators begin the procedure with an angiographic injection in every aortic sinus in order to identify the PVL and determine the relationship with neighboring structures such as the coronary arteries.

**Figure 3:**
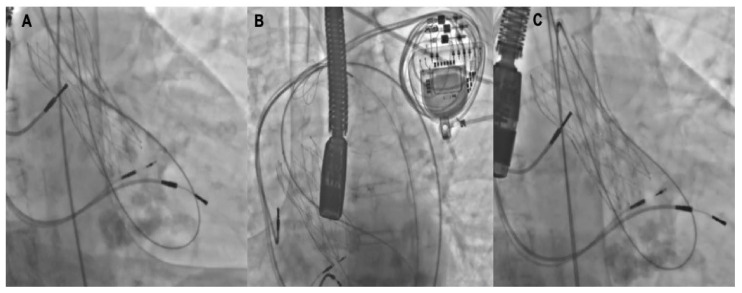
Tips and tricks for support improvement. A 5-Fr Amplatz left catheter (AL-1) (**A**) is across the leak and the 0.035–260 cm hydrophilic guidewire placed in the left ventricle is advanced back through the center of the valve and then snared in the ascending aorta (**B**) and externalized through another arterial access, creating a supportive arterio-aorta rail (**C**) to retrogradely advance the delivery sheath.

After that, selection of the most appropriate shape of the catheter may help to point the catheter towards the leak. In this sense, Judkins Right (JR) or Multipurpose for posterior leaks and Amplatz left catheter (AL-1) for anterior leaks are the most used catheters. Subsequently, hydrophilic wires are generally used to cross the leak. If the hydrophilic wire does not cross, other wires such as coronary wires may ease this process. Once the wire is advanced through the PVL, the diagnostic catheter is advanced through the wire into the LV. Then, a high-supportive wire is advanced into the LV. In this regard, dedicated pre-shaped wires such as SAFARI2 (Boston Scientific) might be very useful to increase support while maintaining wire position and safety. In some cases, when the wire is in the LV, it can be advanced back through the center of the valve and then snared in the ascending aorta and externalized through another arterial access, creating a supportive rail (arterio-arterial loop) to advance the delivery sheath both antegradely or retrogradely (Figure 3).

The delivery catheter is then advanced into the LV and the high-supportive wire is retrieved. Finally, the closure device is advanced through the delivery sheath following the sizing rules previously described. In cases in which catheter progression is not possible, mother and child catheter techniques might be very useful for increasing support. Again, comprehensive knowledge of the catheter length is crucial to avoid useless actions. For example, if a 6-Fr 90 cm Destination™ guiding sheath (Terumo, Europe) needs to be used as a mother catheter to advance a 5-Fr delivery catheter through it, the length of this second catheter should be >100 cm. Similarly, if a 5-Fr 110 cm cannot progress and needs to be used as a mother catheter, a >120 cm catheter will be necessary. The creation of an AV loop is another maneuver that could help in cases in which catheter progression is difficult. In any case, once the delivery catheter is crossed, the closure device is advanced up to the tip of the catheter. The distal disc should be initially delivered within the LV cavity. The platinum markers are located on the long axis of the device (both AVP and PLD) and should be oriented along the long axis of the PVL. This can be performed by rotating the delivery sheath as it is moved back and forth. Actually, this might be difficult to achieve. It is important to avoid a counter-clockwise rotation of the delivery cable as this may result in premature device release when using AVP technology. When the distal disc is in a good position, then the proximal disc is deployed. Before releasing the device, a careful assessment of device positioning with special attention to the interference with neighboring structures such as valve leaflets or coronary arteries is mandatory. Comparison of the maximum and mean aortic gradients before and after device implantation is also recommended to rule out left-ventricle-outflow-tract (LVOT) obstruction. In the absence of any complication and after checking stability, the closure device can be released.

## 5. PVL Regurgitation after TAVI: Procedural Aspects

TAVI is a well-established means for treating patients with severe symptomatic aortic stenosis at high or prohibitive surgical risk. This rapidly evolving technology has recently emerged as a viable alternative, even in patients with intermediate-to-low surgical risk, with bicuspid aortic-valve disease and with pure aortic regurgitation [14].

As TAVI procedures are progressively growing, the need for PVL treatment will become even more frequent. Moderate-to-severe PVL regurgitation (PVLR) after implantation of first-generation transcatheter aortic valves represents a serious complication with adverse impacts on short- and long-term clinical outcomes. [15]. In addition, the higher observed mortality, even in cases with moderate PVL, might lead to a relevant increase in these procedures [16]. In fact, the incidence of moderate or severe PVLR after TAVI ranges from 5% to 25% depending on the valve and the series [17], and has been reported to be higher following the use of self-expandable (SE) valves compared with balloon-expandable (BE) valves [18], probably due to either an inadequate radial strength or an insufficient immediate seal. It is noteworthy to mention that the incidence of PVLR has recently declined with the development of second-generation transcatheter aortic valves, with the incorporation of an outer skirt at the ventricular side of the stent, thereby obtaining better adhesion of the valve at the annular and ventricular levels.

The technological development of third-generation transcatheter valves is mandatory, not only to improve paravalvular sealing with specific skirts and new cuffs in order to better seal the annular region, but also to reduce the risk of stroke, to avoid heart block and the need for pacemaker implantation, and to increase valve durability.

**Figure 4:**
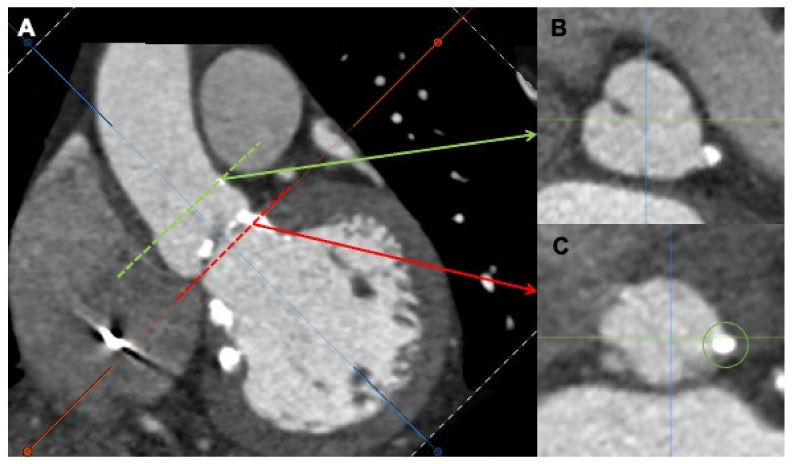
Multidetector computed tomography angiography (MDCTA) showing severe calcification at the level of the left coronary cusp (**A**). Transversal views at the left coronary artery (**B**), green arrow and the aortic ring (**C**), red arrow.

**Figure 5:**
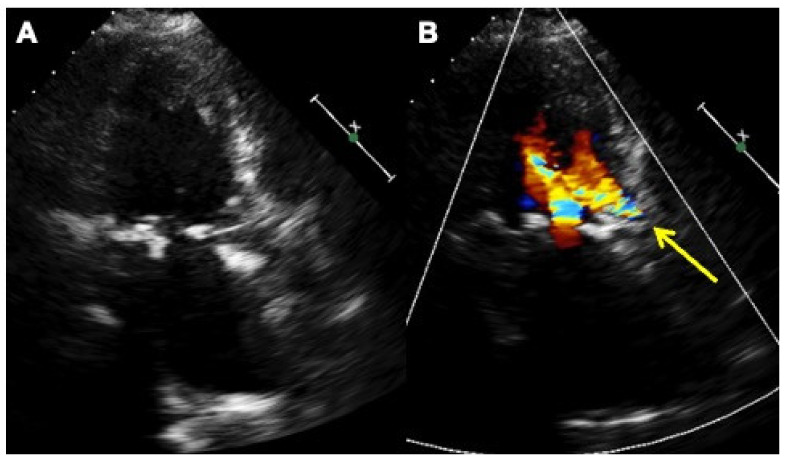
Two-dimensional transthoracic echocardiography color Doppler in two-chambers plane without (**A**) and with color Doppler (**B**) showing the self-expandable aortic biological valve in place, the paravalvular leak at the left coronary aortic cusp with the corresponding regurgitant jet (yellow arrow).

TAVI patients are usually elderly with a high number of comorbidities that include frailty, cognitive impairment, disability and bleeding risk. For these reasons, percutaneous treatment of APVLs might represent an effective and less invasive treatment option for those high-risk patients. Furthermore, multiple or irregular lesions, low crossing positions across frame struts, and high sealing skirts on SE devices are especially challenging. Additionally, aortic-valve calcification plays a role in the onset of PVLR after TAVI (Figure 4 and Figure 5).

**Figure 6:**
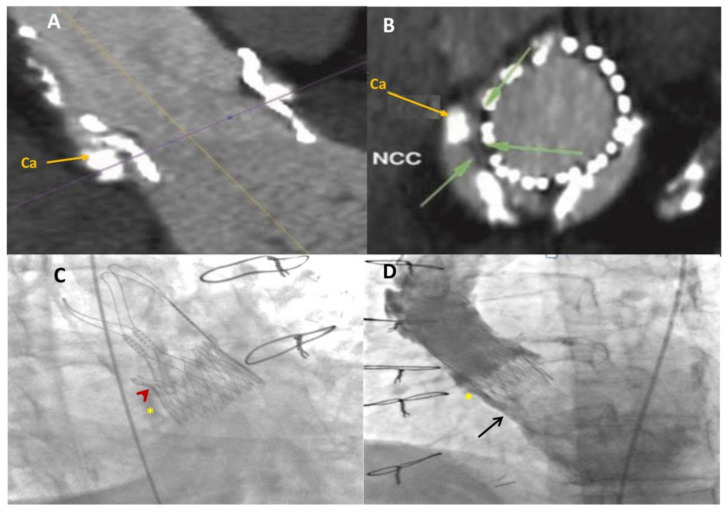
Multidetector computed tomography angiography (MDCTA) scans with acquired frames in the axial (**A**) and coronal (**B**) views showing the location of the leakage (green arrows) and the calcified nodule (Ca, yellow arrow) located at the level of the regurgitant jet at the non-coronary aortic cusp; (**C**) fluoroscopic image showing the self-expandable aortic bioprosthetic valve in place and the calcified nodule (yellow star) and its indentation (red arrowhead) on the non-coronary cusp; (**D**) baseline ascending-aorta angiogram (LAO projection) confirming severe paravalvular regurgitation (black arrow) of the self-expandable bioprosthetic aortic valve in close proximity to the calcified nodule (yellow star) at the non-coronary cusp. NCC, non-coronary cusp; AA, ascending aorta.

In particular, the device-landing-zone calcium volume is associated with PVLR, particularly after SE valves, and the risk increases by 8% every 100 mm^3^ of calcium volume [19] (Figure 6).

Indeed, BE valves seem to be associated with a lower incidence of PVLR when taking valve calcification into account.

In selected cases, the use of a percutaneous radial approach with mild sedation and TTE guidance can be safe and effective [20]. Overall, the procedural steps for APVL closure are the same as in conventional surgical valves. Nonetheless, post-TAVR PVLs might be more challenging to cross and may require catheter progression through the valve struts. Furthermore, angiographic injections in the optimal projection are crucial to identify the path and manipulate the wires to cross the defect.

In general, the treatment of APVLs in SE valves is more challenging than BE valves. BE prostheses are very similar to surgical valves and strut avoidance is generally less challenging. In contrast, SE valves require advancing through the valve struts in most cases. In this regard, catheter and wire manipulation through the valve struts might be very difficult. Mother and child catheter techniques or AV loops are of paramount importance in this setting as delivery catheters are generally difficult to advance. The creation of arterio-arterial or arterio-venous loops through the leak and antegrade progression of delivery catheters from the LV across the PVL may be very helpful to overcome the resistance in order to cross through the struts of the valve from a retrograde approach (Figure 3) [21].

In several cases, dedicated coronary wires are required to cross the leak. The use of more than one coronary wire as a buddy wire may help to advance catheters through the leakage. Another common finding in post-TAVR PVLs is the calcification of the path and therefore the resistance to the advancement of large delivery catheters through the leak. In fact, many reported cases describe the implantation of small or medium closure devices, as large delivery catheters cannot advance through the leak.

## 6. Procedural Risks and Complications

The rate of complications from PVL closure is relatively low, especially when compared to the results of a surgical redo [22]. Periprocedural adverse events may include death, myocardial infarction due to coronary obstruction, AV block, stroke, device embolization, prosthetic leaflet impingement, emergency cardiac surgery, significant pericardial effusion or cardiac tamponade, and bleeding. In the HOLE registry including mitral and aortic PVLs [4], the overall major-adverse-event rate (death, stroke, and emergency surgery) was 4.5%. The most frequent adverse events were vascular complications and bleeding (8.6%). Data comparing aortic and mitral PVLs are scarce, so there are no comprehensive data comparing outcomes between them. Based on the author’s experience, aortic PVLs, as compared to mitral PVLS, seem to be associated with a lower risk of prosthetic leaflet/disc impingement and device embolization. In contrast, there is a potential risk of coronary obstruction [23] and more vascular complications as arterial vascular access is generally mandatory. Final contrast injections to rule out coronary obstruction and baseline (postprocedural measurements of transaortic gradient) are recommended to minimize potential complications. The use of percutaneous vascular closure devices for arterial access is also advised since most patients have mechanical valves and oral anticoagulation needs to be restarted.

## 7. Conclusions

Undoubtedly, APVL closure is a complex and challenging procedure with several periprocedural complications, requiring optimal patient selection and comprehensive cardiac-imaging intraprocedural guidance. Although technically demanding, strategies to increase the success rate of percutaneous closure by expert operators might increase the safety and efficacy of this less invasive treatment option for high-risk patients with symptomatic PVL regurgitation.

The successful PVL reduction to mild or less results in symptomatic acute and long-lasting improvements in clinical parameters, including NYHA class, and reduces the dependency on hemolysis-related blood transfusions and the need to redo cardiac surgery.

Further technological advances such as a lower profile, slippery delivery sheaths and newly designed custom-made devices will also improve procedural success and clinical outcomes. Likewise, future TAVI devices need to be designed to minimize the occurrence of PVL.

## Data Availability

The data presented in this study are contained within the article.

## Supplementary Materials

The following supporting information can be downloaded at: https://www.mdpi.com/article/10.3390/jcm11112989/s1, Supplementary Figure S1. Baseline 2D Transesophageal Echocardiogram (TEE) color Doppler at 50, 125 and 128 degrees showing a huge 16 × 4 mm crescent-shaped noncoronary sinus (NCS) paraprosthetic leakage (orange asterisk) extending towards left coronary sinus (LCS) (**A**) where another 12 × 3 mm oval-shaped leak was present with severe regurgitation (**B**,**C**) in a patient with previous aortic root patch + Aortic Valve Mechanical Replacement (Carbomedics 21) in congestive cardiac failure (NYHA III-IV). Supplementary Figure S2. Fluoro-angiographic procedural steps of the same patient in Supplementary Figure S1. From the left and right femoral arteries two separate 0.035–260 cm stiff guidewires were placed retrogradly into the left ventricle (**A**) and then two simultaneous devices (a 10 × 4 mm-rectangular waist PLD, orange asterisk, and a 7 × 4 mm-rectangular twist PLD, red asterisk) were implanted without impingement on the mechanical prosthetic valve. RW PLD, rectangular waist Occlutech Paravalvular Leak Device; RT PLD, rectangular twist Occlutech Paravalvular Leak Device.
