# Supplementary figures and images for: Paravalvular Leakages after Surgical Aortic-Valve Replacement and after Transcatheter Aortic-Valve Implantation: Strategies to Increase the Success Rate of Percutaneous Closure
Source: J Clin Med. 2022 May 25;11(11):2989. doi: 10.3390/jcm11112989 (PMC9181351; doi:10.3390/jcm11112989)

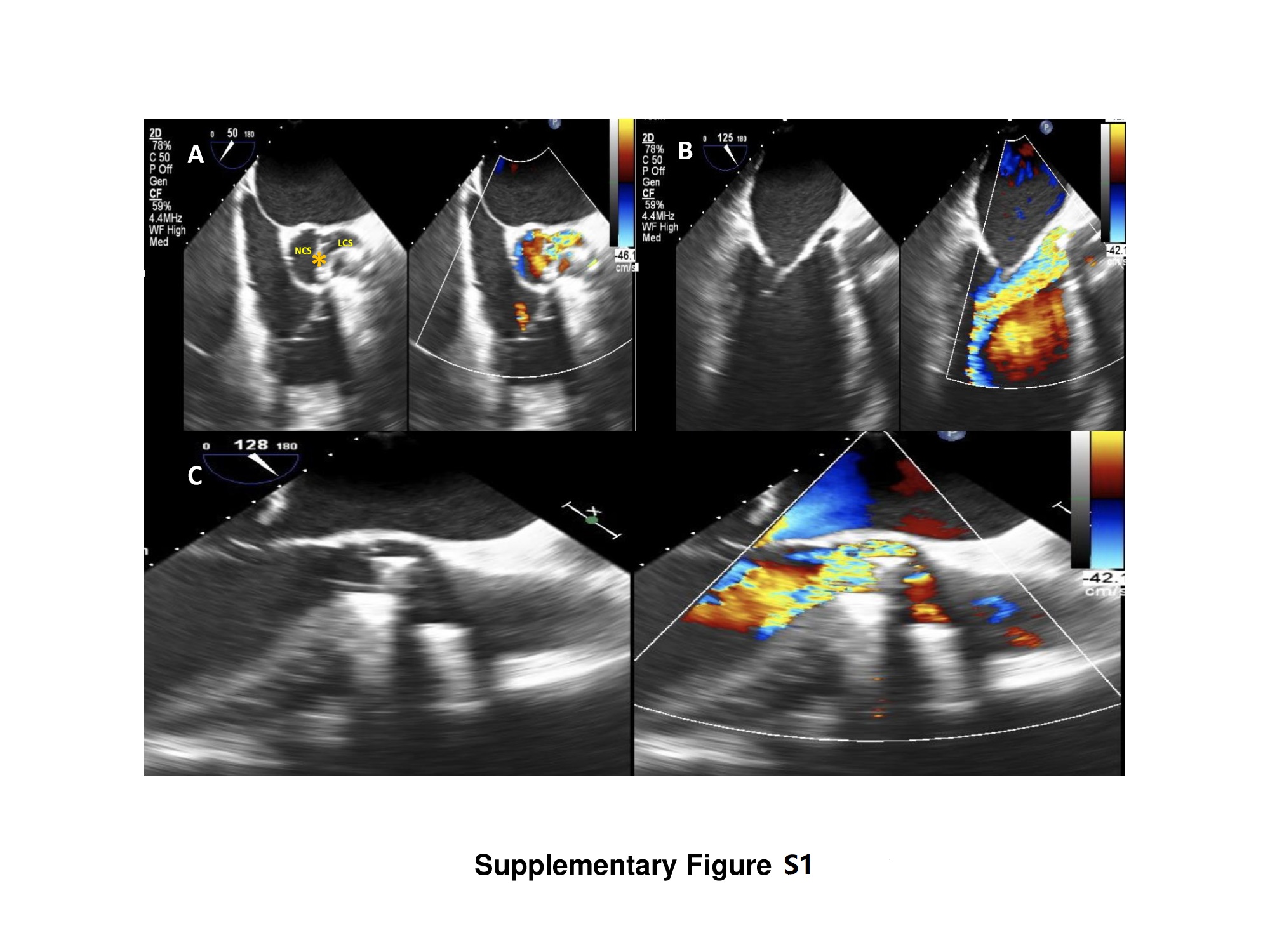

Supplement: Supplementary file 1 [file jcm-11-02989-s001.zip › Supplementary-Figure S1.jpeg]

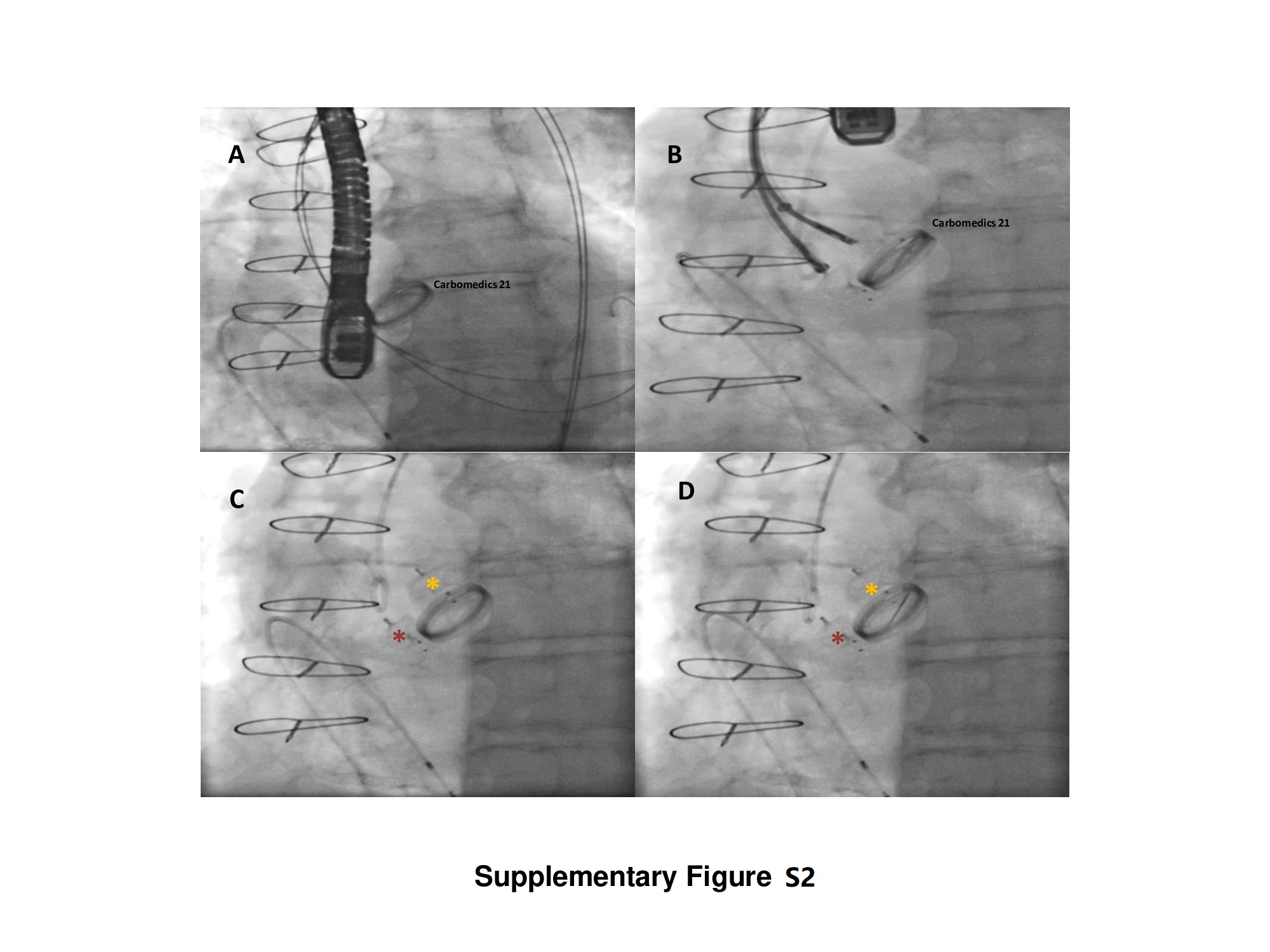

Supplement: Supplementary file 1 [file jcm-11-02989-s001.zip › Supplementary-Figure S2.jpg]
